# Health-related quality of life and treatment satisfaction in Palestinians with rheumatoid arthritis: a cross-sectional study

**DOI:** 10.1186/s41927-022-00251-5

**Published:** 2022-04-06

**Authors:** Heba Abu Hamdeh, Samah W. Al-Jabi, Amer Koni, Sa’ed H. Zyoud

**Affiliations:** 1 Department of Clinical and Community Pharmacy, College of Medicine and Health Sciences, An-Najah National University, Nablus, 44839 Palestine; 2 Division of Clinical Pharmacy, Hematology and Oncology Department, An-Najah National University Hospital, Nablus, 44839 Palestine; 3 Poison Control and Drug Information Center (PCDIC), College of Medicine and Health Sciences, An-Najah National University, Nablus, 44839 Palestine; 4 Clinical Research Centre, An-Najah National University Hospital, Nablus, 44839 Palestine

**Keywords:** HRQoL, Treatment satisfaction, Rheumatoid arthritis, TSQM, Quality of life

## Abstract

**Background:**

Studying health-related quality of life (HRQoL) and treatment satisfaction have helped in understanding how to optimize rheumatoid arthritis (RA) treatment outcomes and find ways to alleviate signs and symptoms among patients.

**Objective:**

In this study, our objective was to evaluate the association between satisfaction with care and HRQoL among RA patients from northern Palestine. In addition, this study also aimed to determine the associations between the clinical characteristics of patients with RA with treatment satisfaction and HRQoL.

**Methods:**

This was a multicenter cross-sectional study conducted between July and October 2018. Patients with RA diagnosis who presented at rheumatology clinics were interviewed. The SF-36 short questionnaire was used to assess HRQoL and Treatment Satisfaction Questionnaire for Medication (TSQM) version 1.4 to assess treatment satisfaction among study groups. We use descriptive and comparative statistics to present the results.

**Results:**

A total of 283 patients were included. Several sociodemographic and clinical characteristics were found to be associated with poor HRQoL scores and low treatment satisfaction. The physical component summary (PCS) was negatively associated with age, patients’ self-reported disease activity, duration of the disease, and the total number of medications taken by the patient, and was positively associated with educational background, employment, and household income. The mental component summary (MCS) was negatively associated with patients’ self-reported disease activity and the patient's total number of comorbid diseases. The number of comorbid diseases was negatively associated with effectiveness. All HRQoL subscales were significantly correlated with treatment satisfaction. The range of correlation with PCS was between 0.272 for convenience and 0.425 for side effects (*p* < 0.001). Similarly, the highest correlation with MCS was 0.458 for side effects, and the lowest was 0.337 for convenience (*p* < 0.001).

**Conclusions:**

The current study found that HRQoL was significantly correlated with treatment satisfaction. Furthermore, the results of this study showed that HRQoL and treatment satisfaction are likely to be affected by sociodemographic and clinical characteristics. These results may be beneficial in clinical practice, mainly in the early treatment of patients with RA, at a stage where it is still possible to increase treatment satisfaction.

**Supplementary Information:**

The online version contains supplementary material available at 10.1186/s41927-022-00251-5.

## Background

Nowadays, the burden of noncommunicable diseases (NCD) has increased in low and middle-income countries (LMICs), while it decreases in high-income countries [1]. These LMICs now have a double of communicable and NCDs [2]. Rheumatoid arthritis (RA) is one of the NCDs that significantly causes morbidity [2]. However, it is still neglected as it is among the main recognized NCDs that contribute to mortality [3]. The burden of RA can be directed through economic expenditures (costs of medication, hospitalization, visits, and caregivers and helpers). The indirect burden of RA can be seen in its negative effect on productivity through absenteeism and early retirement, and intangible costs that are estimated through the impact of the disease on the patient’s quality of life [2].

In LMICs, RA patients face more challenges than those in high-income countries, such as lack of infrastructure, e.g., electricity, hot water, and inadequate public transportation that will force patients to walk longer distances. In addition, patients in LMICs have lower educational levels than those in high-income countries, negatively affecting their psychology and reducing their chances of modifying employment to suit their disabilities. Moreover, the patient will have fewer opportunities to have an active role in problem-solving [4].

In addition, the limited resources in these countries will make it more difficult for patients to access biological treatment or joint replacement surgery. This will cause significant functional disability among these patients, and they will probably lose their employment within two years of symptom onset [4]. For example, the prevalence of functional disability among patients with RA was 72.6% in Kenya [5] and 71% in low-income Hispanic patients [6]. However, it was much lower in a cohort of Olmsted County, Minnesota (26%) [7] and China (15.8%) [8]. Furthermore, a study from Ecuador found that the prevalence of functional disability was 26.6% [9].

In Palestine, the Ministry of Health had a RA management protocol in 2014. Conventional disease-modifying antirheumatic drugs (DMARDs), such as hydroxychloroquine, methotrexate (MTX), leflunomide, are available in Palestine, along with biologic DMARDs (i.e. etanercept, adalimumab, and rituximab). In addition, other medications have been added since 2014, including Tocilizumab, Tofacitinib, and Infliximab. These medications are available in hospitals in Palestine, where outpatients visit rheumatology clinics [10].

In clinical and policy research, the concepts of treatment satisfaction and health-related quality of life (HRQoL) are commonly used to improve pharmaceutical care and treatment outcomes [11–13]. Higher patient treatment satisfaction is correlated with improved HRQoL [14–16]. HRQoL also refers to self-reported physical and mental health measures influenced by individuals’ attitudes, experiences, expectations, and perceptions [17].

Although several studies have been conducted to evaluate the relationship between HRQoL and treatment satisfaction [11, 12, 15, 18–26], their results could not be generalized to the Arab world like Palestine. This is due to the lack of basic resources and healthcare facilities. Consequently, the objectives of the current study were to: (1) evaluate the relationship between treatment satisfaction and HRQoL in RA samples in northern Palestine; (2) assess the impact of sociodemographic and clinical factors on quality of life and treatment satisfaction. Furthermore, it is hoped that treatment satisfaction and HRQoL evaluation offer an opportunity to incorporate patient perspectives into clinical decision-making [27], which can eventually improve treatment outcomes and lower healthcare costs.

## Methods

### Study design and setting

A cross-sectional design was adopted to recruit patients who have been diagnosed with RA according to the 2010 American College of Rheumatology (ACR)/European League against Rheumatism (EULAR) classification criteria [28] were included. The attending rheumatologist at the clinic was responsible for evaluating RA patients according to the ACR/EULAR criteria and evaluating the patient’s clinical status. Two clinical pharmacists collected the data through face-to-face interviews, and the patients were chosen using the convenient sampling method. The study was carried out in rheumatology clinics in Northern West Bank, Palestine. The clinics included in the study were Alwatani Hospital—Nablus, Khalil Suleiman Hospital – Jenin, Thabet-Thabet Hospital—Tulkarem, and Darwesh Nazzal Hospital—Qalqilia. Data were collected between July and October 2018. This study adheres to the STROBE guideline.

### Sample size and sampling technique

According to the previous study [29], approximately 1042 RA patients were referred to rheumatology clinics in Northern West Bank, Palestine, during the year 2012. Therefore, using the proportional quota sampling process, a convenience sample of 281 RA patients was taken to represent the general RA population. Using an automated software program, the appropriate sample size for this analysis was determined (Raosoft sample size calculator: (http://www.raosoft.com/samplesize.html), assuming a confidence interval of 95%, with a 5% margin of error and a 50% response distribution. Furthermore, the target sample size was increased from 5 to 10% to minimize incorrect findings and improve research reliability.

### Inclusion and exclusion criteria

The study included patients older than 18 years of age who were able to provide informed written consent. It excluded those with cognitive impairment or current severe diseases, for example, cancer and stroke. In addition, patients who suffer from other autoimmune rheumatic diseases, chronic inflammatory diseases, neuropsychiatric disorders, juvenile idiopathic arthritis, and fibromyalgia were excluded, as the manifestations and complications of these diseases can affect the patients’ quality of life, consequently, it may create bias in the study findings [28].

### Ethical consideration

This study was approved by the *Institutional Review Board (IRB) of An-Najah National University.* The approval letter was issued on 9 May 2018. They were informed about: 1. The aim and importance of the study; 2. Your confidentiality will be a top priority, as you were identified as numbers marked at the top of the questionnaire, and you were assured that all information would be used only for research purposes only; 3. They were told that they could withdraw from the study anytime they wanted without any consequences.

### Measurements

Demographic characteristics and disease characteristics, age, duration of the disease, gender (male, female), marital state (married, single, divorced or widowed), employment status (employed, unemployed), education (primary education, primary education, junior high school, senior high school college or above), household income (low lower than 400 JD, moderate between 400 and 1000 JD, higher than 1000 JD), residency (rural area, urban area, refugee camp), treatment status (newly diagnosed, regular treatment, non-formal treatment), comorbidities, and prescribed medications were taken using a demographic data questionnaire developed for this study. The medications received were documented (not on any drug, MTX only, MTX + antimalarial, MTX + sulfasalazine, antimalarials only, biological medications) [28]. Furthermore, patients were asked to describe their disease activity at the time of the interview (how would you describe your disease activity: inactive, low to moderate, or high). They need to choose either active (low to moderate, or high) or inactive disease.

### Instruments and data collection forms

Health-related quality of life: In this cross-sectional study, the Rand 36 item short-form Health Survey (SF-36) was used to assess HRQoL [30–34]; The SF-36 is a valid and reliable generic tool that is capable of measuring the impact of disease on the HRQoL, it can also compare healthy and unhealthy populations [31, 32, 34]. The Arabic version of this scale is valid and reliable [35]. Indeed, the validity of the face and content of the Arabic version of this tool was previously discussed, evaluated and used in Palestine [36]. The SF-36 assesses both the physical and psychological domains of HRQoL. It consists of 8 parts; 4 of them will calculate the physical component summary (PCS), which is a combination of the physical functioning (PF), role-physical (RP), bodily pain (BP), and general health (GH). The mental component summary (MCS) was calculated by summarizing the other four parts, and they are vitality (V), social functioning (SF), role-emotional (RE), and mental health (MH) [28, 30, 31, 37, 38]. The scores were summed, and the results ranged from 0 to 100, where 0 indicates the worst health status and 100 indicates the best health status. The scoring algorithm was applied to obtain both the PCS and MCS [28]. A Cronbach’s alpha was calculated for the scores in the eight domains to calculate reliability. In the current study, Cronbach's alpha for all subscales ranged from 0.672 to 0.734, indicating an acceptable level of internal consistency of the study questions.

Treatment satisfaction was measured using an Arabic version of the Treatment Satisfaction Questionnaire for Medication (TSQM 1.4) that contains 14 items that measured four domains which are effectiveness (questions 1–3), side effects (questions 4–8), convenience (questions 9–11) and overall satisfaction (questions 12–14) [12, 26, 39, 40]. The Arabic version of TSQM 1.4 is a valid and reliable tool to assess treatment satisfaction [41], and was previously used in many publications in Palestine [18, 26, 39]. Its score ranges from 0 to 100; higher scores indicate higher treatment satisfaction. An-Najah National University has been approved to use this questionnaire by IQVIA™.

### Pilot study

A pilot study (6 participants) has been conducted to ensure the availability of the required data and estimate the time needed for the interview. The final report did not include the patients involved in the pilot study.

### Statistical analysis

Data were analyzed using the Statistical Package for Social Sciences program version 15 (SPSS).

General and baseline characteristics between individuals were reported using mean (standard deviation (SD)) or S medians (lower–upper quartiles) for continuous variables and frequency and percentages for categorical variables. Variables were tested using the Kolmogorov–Smirnov test for normality. Kruskal–Wallis or Mann–Whitney tests were used to search for variations between groups in mean rank and median [interquartile range]. Furthermore, to determine the correlation between the reported SF-36 scores and the TSQM scores, Spearman's correlation coefficient was used. The significance level was established at a p-value < 0.05.

## Results

### Sociodemographic characteristics of the sample

Table 1 shows the sociodemographic characteristics of the sample, which consisted of 285 patients with RA from 4 hospitals in the northern area in the West Bank. Most of the sample was female (231, 81.1%), so the female: male ratio is 4.3:1. The mean (± SD) age of the patients was 52.0 ± 13.7, ranging from 18 to 86 years old.

**Table 1:** Sociodemographic characteristics of the study group

Variable		Frequency (%) N = 285 Or Mean ± SD
Hospital	Qalqilia	39 (13.7)
	Tulkarm	70 (24.6)
	Jenin	87 (30.5)
	Al Watani	89 (31.2)
Gender	Male	54 (18.9)
	Female	231 (81.1)
Age (Years)		51.95 ± 13.73
Age Group	Less than 30	21 (7.4)
	30 years—39 years	26 (9.1)
	40 years—49 years	69 (24.2)
	50 years—59 years	87 (30.5)
	≥ 60	82 (28.8)
Smoking	Smoker	50 (17.5)
	Non-smoker	235 (82.5)
Educational level	Below Primary Education	13 (4.6)
	Primary Education	57 (20)
	Junior High School	73 (25.6)
	Senior high School	57 (20)
	Collage or more	85 (29.8)
Marital status	Single	53 (18.6)
	Married	199 (69.8)
	Divorced/ Widowed	33 (11.6)
Employment	Employed	67 (23.5)
	unemployed	199 (69.8)
	Stopped because of RA	19 (6.7)
Place of residence	City	101 (35.4)
	Village	169 (59.3)
	Refugee Camp	15 (5.3)
Household income	Low: Less than 400 JD	145 (50.9)
	Moderate: Between 400–1000 JD	119 (41.8)
	High: More than 1000 JD	20 (7)
Body mass index		28.788 ± 5.65
Body mass index category	Underweight / Normal	52 (18.2)
	Overweight	129 (45.3)
	Obese	104 (36.5)

1 Jordanian dinar (JD) equals 1.41 US dollar

### Clinical characteristics of RA patients

Table 2 presents the clinical characteristics of patients with RA. Regarding patients’ self-reported disease activity, 43.5% of patients had inactive disease. The mean duration of the disease (± SD) was 9.1 ± 8.2. Regarding the treatment status, the majority of them (239, 83.9%) were on regular treatment, while 46 (16.1%) patients had non-formal treatment. In addition, Table 2 shows the comorbidities these patients have. The most prevalent comorbidity among the study group was hypertension. Hypertensive patients were 97 (34.0%), followed by diabetes 52 (18.2%), heartburn 25 (8.8%), and constipation 24 (8.4%).

**Table 2:** Clinical characteristics of patients

Variable		Frequency (%) N = 285 Or Mean ± SD
Patient self-reported disease activity	Inactive	124 (43.5)
	Low to moderate	98 (34.4)
	High	61 (21.4)
Status of treatment	Regular treatment	239 (83.9)
	Non formal treatment	46 (16.1)
Duration of disease (years)		9.06 ± 8.21
Duration of the disease	< 1	9 (3.2)
	1–3 years	73 (25.6)
	4–5 years	52 (18.2)
	> 5 years	150 (52.6)
Comorbidities	Hypertension	97 (34%)
	Diabetes	52 (18.2%)
	Heart burn	25 (8.8%)
	Constipation	24 (8.4)
	Disk displacement	23 (8.1%)
	Irritable bowel disease	21 (7.4%)
	Eye dryness	18 (6.3%)
	Cholecystectomy	16 (5.6%)
	Osteoporosis	12 (4.2%)
Total number of Comorbid diseases		1.65 ± 1.78
Total number of comorbid diseases	Zero	91 (31.9)
	One Comorbid Disease	69 (24.2)
	Two Comorbid Disease	53 (18.6)
	Three Comorbid Disease	37 (13)
	≥ 4 Comorbid Disease	35 (12.3)
Total number of medications		6.54 ± 3.44
Total number of medications	1–3 medications	41 (14.4)
	4–6 medications	122 (42.8)
	≥ 7	122 (42.8)
Total number of RA medications		2.03 ± 0.853

### Medications and management of the rheumatoid arthritis

Table 3 shows the medications prescribed to the patients. In more detail, patients were categorized into 3 groups, a high percentage of them were prescribed 4–6 medications, and seven medications or more were 122 (42.8%). The range of medications prescribed to patients ranged from 1–30 medications. The number of RA medications prescribed to patients ranged from 0 to 5. The mean (± SD) was 2.0 ± 0.9. Paracetamol was the predominant analgesic used (38.2%). Of the NSAIDs, the most prescribed was diclofenac sodium (19.6%). For corticosteroids, prednisolone was the main prescribed medication (57.2%). For RA medications, methotrexate was prescribed to the majority of patients; they were 169 (59.3%) patients, while for biological drugs, it was Etanercept, which was prescribed to a fifth of patients, 61 (21.4%).

**Table 3:** Prescribed medications currently used by RA patients

RA medications	Frequency (%) N = 285
Paracetamol	109 (38.2%)
Paracetamol + Orphenadrine citrate	15 (5.3%)
Ibuprofen	38 (13.3%)
Diclofenac Sodium	56 (19.6%)
Etoricoxib	16 (5.6%)
Etodolac	2 (0.7%)
Meloxicam	41 (14.4%)
Nimesulide	2 (0.7%)
Prednisolone	163 (57.2%)
Methotrexate	169 (59.3)
Sulfasalazine	18 (6.3%)
Hydroxychloroquine	70 (24.6%)
Leflunomide	83 (29.1%)
Etanercept	61 (21.4%)
Adalimumab	8 (2.8%)
Rituximab_Mebthera	7 (2.5%)

### Frequencies of SF-36

More than a third of the patients (106; 37.2%) considered their health good, while 80 (28.1%) answered that their health now is somewhat better than one year ago. For routine daily life activities, 196 (68.8%) patients were greatly limited in vigorous activities and 109 (38.2%) had many limitations in lifting or carrying groceries.

### Sociodemographic and clinical characteristics of PCS and MCS subscales

Tables S1 and S2 show the sociodemographic and clinical characteristics of patients with PCS and MCS subscales relationships. We performed the Kruskal–Wallis test and Mann–Whitney to test for differences in means between categories (Additional file 1). Age was negatively associated with physical Functioning scores (*p* < 0.001), the median (IQR) of patients aged 40 to 49 years old was the highest 55.0 [31.3–68.8].

Education was positively associated with physical functioning (*p* = 0.009), the median (IQR) of those in junior and senior high school had higher scores than others, they were 50.0 [35.0–60.0] and 50.0 [30.0–70.0], respectively. Education was also positively associated with role limitation due to physical functioning (*p* = 0.020); the median was zero. Social functioning was also significantly associated with education (*p* = 0.016), the median (IQR) for junior, senior high school, and college or more were 50.0 [37.5–84.4], 50.0 [37.5–75.0], and 50.0 [37.5–75.0] respectively, and finally education was positively associated with bodily pain (p < 0.001), where the median (IQR) for both senior high school and college or more groups was 45.0 [32.5–57.5] and 45.0 [32.5–47.5], respectively.

Regarding employment, it was positively associated with physical functioning (*p* < 0.001), where the median (IQR) of employed patients was the highest 55.0 [42.5–75.0]. Furthermore, it was positively associated with limitation of role due to physical functioning. The median of all categories was zero and it was positively related to general health (*p* = 0.015), where the median (IQR) of employed patients was the highest 45.0 [28.8–56.3].

The place of residence was only significantly associated with RP; the median of all was zero; urban areas were positively affected. Household income was positively associated with PF (*p* < 0.001), where the highest median IQR was for those whose income was more than 1000 JD 55.0 [40.0–85.0]. Furthermore, it was positively associated with RP (*p* = 0.003), the highest median (IQR) was also for those whose income is greater than 1000 JD. Moreover, it was positively associated with VT (*p* = 0.038), the median (IQR) was highest for both moderate and high-income people, 45.0 [30.0–56.3] and 45.0 [41.3–48.8], respectively. Moreover, it was positively associated with MH where (*p* = 0.014), with the highest median (IQR) for those with high income of 74.0 [59.0–86.0]. BP and GH were positively associated with household income, where the *p* values were 0.012 and 0.002, respectively, and the median (IQR) was highest for those with moderate income, 45.0 [35.0–57.5] and 45.0 [30.0–50.0].

BMI was only significantly associated with GH (*p* = 0.023), and the highest median was for those who were overweight, 45.0 [30.0–53.8]. Patients’ self-reported disease activity was negatively associated with the eight scores with PF (p < 0.001) and the highest median (IQR) for those with inactive disease, with RP (*p* < 0.001) and the highest median (IQR) was zero for all, with RE (*p* < 0.001) and highest median (IQR) for those with inactive disease 33.3 [0.0–100.0], with VT (*p* < 0.001) and the highest median (IQR) was 45.0 [35.0–55.0] for those with inactive disease, with MH (*p* = 0.001) with the highest median (IQR) 60.0 [44.0–78.0] for those with inactive disease, with SF (*p* = 0.002) with the highest median (IQR) for both inactive disease and low to moderate disease activity 50.0 [37.5–75.0] and 50.0 [37.5–84.4], respectively. Patients’ self-reported disease activity was also negatively associated with BP (*p* < 0.001), and the highest median was for those with inactive disease 45.0 [32.5–61.3]. And finally, with GH (*p* < 0.001) and the highest median was for those with inactive disease 50.0 [35.0–60.0].

The duration of the disease was negatively associated with RP (*p* = 0.007) and GH (*p* = 0.026). The total number of comorbid diseases that the patient has was negatively associated with all dimensions except MH. The total number of medications taken by the patients was also negatively associated with 4 SF dimensions; they are PF (*p* < 0.001), RP (*p* = 0.001), BP (*p* = 0.010) and GH (*p* = 0.021).

### Sociodemographic and clinical characteristics of PCS and MCS

Table S3 shows the results of the Kruskal–Wallis test or the Mann–Whitney test for differences in means between categories (Additional file 1). PCS was negatively associated with age (*p* = 0.007), patients’ self-reported disease activity (*p* < 0.001), duration of disease (*p* = 0.018), and the total number of medications taken by the patient (*p* < 0.001), and was positively associated with educational background (*p* < 0.001), employment (*p* = 0.001), and household income (*p* < 0.001). MCS was negatively associated with patients’ self-reported disease activity (*p* < 0.001), and the total number of comorbid diseases that the patient has (*p* < 0.001).

### Treatment satisfaction among RA patients

Treatment satisfaction was measured with TSQM, consisting of four domains, effectiveness, side effects, convenience, and overall satisfaction. In the first domain effectiveness, the mean ± SD was 60.3 ± 16.7, and the range was between (0.0–100.0), with a median (IQR) of 61.1 (50.0–72.2). In second domain, which is the side effects domain, the mean ± SD was 46.9 ± 25.1, and the range was between (0.0–100.0), with a median (IQR) of 50.0 (31.3–62.5). In the third domain, which is convenience, the mean ± SD was 59.5 ± 14.5, and the range was between 5.56 and 100 with a median (IQR) 61.1 (50.0–66.7). For the fourth domain, which is overall satisfaction, the mean ± SD was 54.9 ± 20.9, and the range was between 8.3 and 100.0 with a median (IQR) of 54.2 (37.5–69.4).

### Sociodemographic and clinical characteristics associated with treatment satisfaction

Table S4 shows the sociodemographic and clinical characteristics of the study group with differences in treatment satisfaction scores (Additional file 1). Side effects were positively associated with household income; the median was the highest for those greater than 1000 JD 56.3 [37.5–93.8] with a *p*-value = 0.016. Although for patients’ self-reported disease activity, the four domains were negatively associated with it, the highest median was for those who had inactive disease in all four domains. For effectiveness, its median was 61.1[50.0–72.2] with a *p*-value < 0.001, for the domain of side effect, they had 56.3 [37.5–68.8] with a *p*-value = 0.004, for convenience, its median was 61.1 [50.0–66.7] with a *p*-value = 0.001, and for overall satisfaction, its median was 59.7 [45.8–69.4] with a *p*-value < 0.001.

Comorbid diseases were negatively associated with effectiveness. Those who had zero or only one comorbid disease had a higher median than the others in the effectiveness domain, and the median was 61.1 [55.6–72.2] and 61.1 [50.0–77.8], respectively, the *p*-value was 0.006.

### Relationship between HRQoL and treatment satisfaction

There is a modest positive correlation between all HRQoL subscales and treatment satisfaction domains (Table 4). The range of the correlation coefficient ranged from effectiveness from (0.263–0.384), side effects (0.273–0.458), convenience (0.294–0.337), and all-over satisfaction (0.391–0.456).

**Table 4:** Correlations between HRQoL subscales and treatment satisfaction

HRQoL subscales	Spearman's rho	Effectiveness	Side effects	Convenience	Overall satisfaction
Physical Functioning	Correlation Coefficient	0.274***	0.370***	0.177***	0.322***
Role-Physical	Correlation Coefficient	0.292***	0.283***	0.211***	0.308***
Bodily Pain	Correlation Coefficient	0.263***	0.379***	0.234***	0.270***
General Health	Correlation Coefficient	0.375***	0.365***	0.299***	0.468***
Energy-Fatigue	Correlation Coefficient	0.384***	0.378***	0.309***	0.462***
Social Functioning	Correlation Coefficient	0.264***	0.416***	0.243***	0.348***
Role-Emotional	Correlation Coefficient	0.295***	0.364***	0.272***	0.340***
Mental Health	Correlation Coefficient	0.312***	0.273***	0.294***	0.391***
Physical Component Summary	Correlation Coefficient	0.347***	0.425***	0.272***	0.390***
Mental Component Summary	Correlation Coefficient	0.372***	0.458***	0.337***	0.456***

^***^ *P* value < 0.001

## Discussion

This study aimed to examine the effect of RA on HRQoL by using the SF-36 tool and the satisfaction with treatment by TSQM. The results of our study showed that RA negatively affects HRQoL; RA affects the physical component more than the mental component, suggesting that RA has a greater impact on the physical component than the mental one. This is consistent with other studies [28, 31, 37, 42]. In the mental component summary MCS, it was only significant with patients’self-reported disease activity and the total number of comorbid diseases the patient has, while in the physical component summary PCS, it was significant with age group, education level, employment, household income, patients’self-reported disease activity, duration of the disease, and the total number of medications taken by the patient. Therefore, it is clear that many sociodemographic and clinical factors affect the physical component of HRQoL, while the mental component is only affected by certain clinical variables. For example, clinicians should try to keep RA patients in a stable condition by selecting the appropriate management, as this factor is associated with both aspects of HRQoL.

Males had better RP than females, which was contradictory to other findings [37], maybe because females in the Arab world live in a very traditional environment, where they have to carry out all household courses without the help from their husband, son, father, or brothers, which in return increase the burden on them and could negatively affect their RP compared to males. Males also had better mental health than females, which was consistent with other studies [37]. In our study, there were sex-related differences in RP; women had a lower score than men, which was consistent with a previous study [32].

Old age was negatively affecting HRQoL-PCS, which follows the findings of other papers [31, 37, 43]. It was noticed that age group ≥ 50 had lower PF compared to other categories; this might be the result that with age, the PF starts to decline [44].

Our study showed that patients with higher educational levels have better HRQoL-PCS, which was also reported by other studies [32, 43]. This is because educated people can better understand their disease, which enables them to control enabling situations much better than others. According to the level of education, it affected positively physical functioning, PF, and bodily pain, BP which was reported by other researchers [45] and role-physical altogether, so lower levels of education were related to lower levels of the three subscales, which in return affected physical HRQoL. On the other hand, higher education positively affected the social functioning of SF. This was in contrast to what other studies found, in which educated people had lower HRQoL in both physical functioning (PF), PCS, and MCS, even though they had less bodily pain than uneducated patients [28], while some studies found that there is no relation between physical HRQoL and attained education [33].

In our results, the employed patients had better physical functioning PF, role-physical RP, and general health GH. Employment was associated with higher physical HRQoL-PCS, following other research [31, 43]. This might be due to the fact that work provides them with a better economic and social status and interpersonal relationships, which will, in return, help them cope with the disease.

The place of residence affected the role-physical, so those who live in cities had a better role-physical RP than those who lived in rural or camp areas, which was in agreement with other researches where rural areas were negatively linked to HRQoL [31]. This could be linked to the fact that those who live in rural areas are probably farmers and perform harder tasks than those who live in cities. On the contrary, for those who live in areas of Palestinian refugee camp, this could be because they also have a harsher daily life than those in cities. In fact, Palestinian refugees complained of very low family income and poor female health, along with living in an overcrowded and unsanitary place [46].

Socioeconomic status SES is underrepresented in research samples worldwide, although they are subjected to increased susceptibility to RA and reduced HRQoL, and they use only a single measure of educational attainment or household income of monthly income to represent the SES of the patient [37]. Our research found that household income affects physical HRQoL, so those with higher income had better PCS than others. On the other hand, they had better physical functioning and role-physical, but they had better mental health and vitality than others. When it came to bodily pain and general health, those with moderate income had better outcomes than others.

There was an inverse correlation between patients’ self-reported disease activity on PCS, MCS, and all HRQoL subscales, so those with severe disease had worse physical and mental health than the others and worse HRQoL. This information extended the information in the literature [42]. Having higher disease activity could be a reason for late diagnosis, lack of aggressive treatment, and self-management. All of this will greatly affect HRQoL. In light of what was mentioned, we must emphasize the importance of regular disease treatment and management.

The duration of the disease affected PCS and RP. Those who had RA for 1 to 3 years had the best PCS, while for general health (GH), those who had RA for less than one year had better GH than others. This is likely to be a reason that, after years of being sick with RA, the disease may progress, which will cause PCS, RP, and general health GH to decline, especially when treatment is not well managed.

Coexisting comorbidities can affect RA outcomes, such as physical health and general health, so it is important to evaluate comorbidities in the research [45], which was the case in our study. All SF-36 subscales except mental health (MH) were affected, so those who had zero comorbidity had better subscales, except in the role-emotional (RE) and general health (GH) roles, which the latter was surprisingly better in those with one comorbid disease. This result could be due to the sample size (statistical type 2 errors). Furthermore, mental health (HRQoL) was better in those with less chronic disease.

Finally, the number of prescribed medications affected PCS, so those who were prescribed fewer medications had better PCS, and physical functioning (PF), role physical (RP), bodily pain (BP), and general health (GH) were better among those who were prescribed fewer medications.

Satisfaction was only affected by a few factors; the first one is the household income, so those with higher income reported fewer side effects than others. Furthermore, it was affected by patients’ self-reported disease activity, which affected all satisfaction domains. Those patients with inactive disease had better effectiveness, fewer side effects, and more convenient treatment for them, and their general satisfaction was much better than others. Finally, the satisfaction with the medication was affected by the number of comorbidities the patient had; those who had no comorbidities reported the best effectiveness of their treatment, among others. However, this depends on the treatment used, as patients with comorbidities can limit treatment options and subsequently exclude the possibility of controlling disease activity.

In our study, a low positive correlation was found between HRQoL and treatment satisfaction. Similarly, previous articles with the same concept and different population groups (diabetes and hypertension) reported a low correlation between HRQoL and treatment satisfaction [14, 18, 26, 47]. It seems that the two scales are somehow different in what they measure [14]. Furthermore, treatment satisfaction is affected by clinician attitudes and the degree of connection with patients [14, 48], where HRQoL can be related to treatment satisfaction as a result of the patient’s attitude of the patients to take their drugs [49]. In other studies, treatment satisfaction was closely related to high patient participation in his health care, including the patient in decision making [50–52], which will increase the patient’s confidence [53].

In addition, it will improve the patient’s adherence to therapy [54] that we need to provide the patient with information from his attending physician, and these efforts should be directed at those with low education, chronic physical disorder, and emotional distress [50]. Other studies also showed that providing the patient with information on the side effects of their medications and treatment options was significantly associated with higher overall satisfaction levels [55]. The beliefs and attitudes of patients influence how they take medication, so healthcare professionals can facilitate the patient’s acceptance of the risk of treatment by clarifying the consequences of side effects, which will alleviate fear inside the patient [52].

The current findings could be generalized to other parts of the West Bank, Palestine, as this area has the same protocol, availability of medications, and accessibility to treatment. However, the results may not represent other parts of Palestine like the Gaza Strip, as this area has different regulations and restrictions.

### Strengths and limitations

This is the first cross-sectional study in Palestine that explored and reported both HRQoL and satisfaction with the medication among RA patients to the best of our knowledge. This study included a sample from all hospitals in northern Palestine, which will create a database for RA disease in Palestine. The data was collected through face-to-face interviews, which will ensure complete data. The most important limitation is that our sample was convenient from 4 hospitals in the West Bank. Moreover, the sample size is small, so generalizability is limited. This is a cross-sectional study, so we cannot establish a causal relation. Our study also lacks a measuring tool for disease activity, which will better evaluate the patient’s situation. The sample size is a major determinant of the risk of reporting false-negative findings (Type II error). Furthermore, marital status was not evaluated in the analysis, which is a significant factor that can affect mental health. And certain sociodemographic characteristics were not well distributed among the categories (that is, almost 80% of patients have a BMI of more than 25, which can directly affect their physical function). Information such as doses of prescribed medications was not collected, and multivariate analysis of the data was not performed.

## Conclusions

In general, the physical HRQoL of RA patients is affected more than the mental one. Gender, age, BMI, education, employment, place of residence, household income, duration and activity of the disease, number of comorbid diseases, and number of medications taken by the patient are all factors affecting HRQoL of RA patients. The satisfaction of the medication is positively affected by HRQoL. The present study raises the importance of income on physical HRQoL, so this finding has important implications for developing a plan to help poor patients by supporting them financially by the government, which will significantly improve their physical HRQoL. However, this study helps us understand the importance of involving RA patients in decision-making, giving them more information about the disease, medications, and adverse effects. In addition, we provide special attention to the elderly and uneducated people who will probably suffer. These findings have important implications: the importance of the role of the multidisciplinary team in educating patients about their disease, medications, and their adverse effects. It is also important to apply a multidisciplinary approach that aims to improve the physical and psychological health of the patient. These interventions will provide individualized management through the rheumatologist and clinical pharmacists, psychologists, physiotherapists, etc.

## Supplementary Information

**Additional file 1. Supplemental Table S1-S4: Supplemental Table S1**: PCS and MCS subscales with socio-demographic and clinical characteristics; **Supplemental Table S2**: Mean rank of HRQoL subscales with socio-demographic and clinical characteristics; **Supplemental Table S3**: PCS and MCS with socio-demographic and clinical characteristics; **Supplemental Table S4**: Treatment satisfaction with socio-demographic and clinical characteristic.

## Data Availability

All datasets collected and analyzed in this survey will be available from the corresponding author upon reasonable request.

## Abbreviations

ACR: American College of rheumatology
bDMARDs: Biological disease-modifying anti-rheumatic drugs
BMI: Body mass index
BP: Bodily pain
cDMARDs: Conventional disease-modifying anti-rheumatic drugs
CVD: Cardiovascular diseases
EULAR: European league against rheumatism
GH: General health
HRQoL: Health-related quality of life
JD: Jordanian dinar
LMIC: Low middle-income countries
MCS: Mental component summary
MH: Mental health
NCD: Non-communicable disease
PCS: Physical component summary
PF: Physical functioning
RA: Rheumatoid arthritis
RE: Role-emotional
RPL: Role-physical
SDL: Standard deviation
SES: Socioeconomic status
SF: Social functioning
SF-36: Short form-36 questionnaire
TSQM 1.4: Treatment Satisfaction Questionnaire for Medication version 1.4
VT: Vitality

## References

[1] Allen L, Williams J, Townsend N, Mikkelsen B, Roberts N, Foster C, Wickramasinghe K (2017). Socioeconomic status and non-communicable disease behavioural risk factors in low-income and lower-middle-income countries: a systematic review. Lancet Glob Health.

[2] Rudan I, Sidhu S, Papana A, Meng SJ, Xin-Wei Y, Wang W, Campbell-Page RM, Demaio AR, Nair H, Sridhar D (2015). Prevalence of rheumatoid arthritis in low- and middle-income countries: a systematic review and analysis. J Glob Health.

[3] Smolen JS, Aletaha D, McInnes IB (2016). Rheumatoid arthritis. Lancet.

[4] Hodkinson B, Tikly M, Adebajo A (2014). Rheumatoid arthritis in the developing world: stepping up to the challenge. Clin Rheumatol.

[5] Odundo B, Ogola E, Oyoo G, Genga E (2019). Prevalence of functional disability in patients with rheumatoid arthritis attending the rheumatoid outpatient clinic at Kenyatta National Hospital. Afr J Rheumatol.

[6] Karpouzas GA, Draper T, Moran R, Hernandez E, Nicassio P, Weisman MH, Ormseth S (2017). Trends in functional disability and determinants of clinically meaningful change over time in hispanic patients with rheumatoid arthritis in the US. Arthritis Care Res (Hoboken).

[7] Myasoedova E, Davis JM, Achenbach SJ, Matteson EL, Crowson CS (2019). Trends in prevalence of functional disability in rheumatoid arthritis compared with the general population. Mayo Clin Proc.

[8] Ji J, Zhang L, Zhang Q, Yin R, Fu T, Li L, Gu Z (2017). Functional disability associated with disease and quality-of-life parameters in Chinese patients with rheumatoid arthritis. Health Qual Life Outcomes.

[9] Intriago M, Maldonado G, Guerrero R, Moreno M, Moreno L, Rios C (2020). Functional disability and its determinants in ecuadorian patients with rheumatoid arthritis. Open Access Rheumatol.

[10] Palestinian Ministry of Health. General Administration of pharmacy. Drug Information Department. 2014. http://pharmacy.moh.ps/Content/PDF/pYExMo4dZ3x1CH2EdwRmdQYT_CbnRPJPRiaoTosdycaKpbBgr.pdf (accessed September 19 2021).

[11] Chen H, Rosenzweig EB, Gotzkowsky SK, Arneson C, Nelsen AC, Bourge RC (2013). Treatment satisfaction is associated with improved quality of life in patients treated with inhaled treprostinil for pulmonary arterial hypertension. Health Qual Life Outcomes.

[12] Atkinson MJ, Kumar R, Cappelleri JC, Hass SL (2005). Hierarchical construct validity of the treatment satisfaction questionnaire for medication (TSQM version II) among outpatient pharmacy consumers. Value Health.

[13] Jiang N, Yang P, Liu S, Li H, Wu L, Shi X, Fang Y, Zhao Y, Xu J, Jiang Z (2020). Satisfaction of patients and physicians with treatments for rheumatoid arthritis: a population-based survey in China. Patient Prefer Adherence.

[14] Redekop WK, Koopmanschap MA, Stolk RP, Rutten GE, Wolffenbuttel BH, Niessen LW (2002). Health-related quality of life and treatment satisfaction in Dutch patients with type 2 diabetes. Diabetes Care.

[15] Nicolucci A, Cucinotta D, Squatrito S, Lapolla A, Musacchio N, Leotta S, Vitali L, Bulotta A, Nicoziani P, Coronel G (2009). Clinical and socio-economic correlates of quality of life and treatment satisfaction in patients with type 2 diabetes. Nutr Metab Cardiovasc Dis.

[16] Bradley C, de Pablos-Velasco P, Parhofer KG, Eschwege E, Gonder-Frederick L, Simon D (2011). PANORAMA: a European study to evaluate quality of life and treatment satisfaction in patients with type-2 diabetes mellitus–study design. Prim Care Diabetes.

[17] Chen H, Taichman DB, Doyle RL (2008). Health-related quality of life and patient-reported outcomes in pulmonary arterial hypertension. Proc Am Thorac Soc.

[18] Al-Jabi SW, Zyoud SH, Sweileh WM, Wildali AH, Saleem HM, Aysa HA, Badwan MA, Awang R (2015). Relationship of treatment satisfaction to health-related quality of life: findings from a cross-sectional survey among hypertensive patients in Palestine. Health Expect.

[19] Benzimra M, Bonnamour B, Duracinsky M, Lalanne C, Aubert JP, Chassany O, Aubin-Auger I, Mahe I (2018). Real-life experience of quality of life, treatment satisfaction, and adherence in patients receiving oral anticoagulants for atrial fibrillation. Patient Prefer Adherence.

[20] Chaturvedi R, Desai C, Patel P, Shah A, Dikshit RK (2018). An evaluation of the impact of antidiabetic medication on treatment satisfaction and quality of life in patients of diabetes mellitus. Perspect Clin Res.

[21] Granado-Casas M, Martinez-Alonso M, Alcubierre N, Ramirez-Morros A, Hernandez M, Castelblanco E, Torres-Puiggros J, Mauricio D (2017). Decreased quality of life and treatment satisfaction in patients with latent autoimmune diabetes of the adult. PeerJ.

[22] Khanna PP, Shiozawa A, Walker V, Bancroft T, Essoi B, Akhras KS, Khanna D (2015). Health-related quality of life and treatment satisfaction in patients with gout: results from a cross-sectional study in a managed care setting. Patient Prefer Adherence.

[23] Masaki S, Tatsukawa R, Uryu M, Takahara M, Furue M, Ohata C, Nakama T, Hino R, Nakamura M, Nakayama J (2017). Treatment satisfaction, willingness to pay and quality of life in Japanese patients with psoriasis. J Dermatol.

[24] Peach G, Romaine J, Holt PJ, Thompson MM, Bradley C, Hinchliffe RJ (2016). Quality of life, symptoms and treatment satisfaction in patients with aortic aneurysm using new abdominal aortic aneurysm-specific patient-reported outcome measures. Br J Surg.

[25] Ramu J, Chatziralli I, Yang Y, Menon G, Bailey C, Eckstein M, Hykin P, Sivaprasad S (2017). Health-related quality of life, visual function and treatment satisfaction following intravitreal dexamethasone implant for diabetic macular edema. Patient Prefer Adherence.

[26] Zyoud SH, Al-Jabi SW, Sweileh WM, Arandi DA, Dabeek SA, Esawi HH, Atyeh RH, Abu-Ali HA, Sleet YI, Abd-Alfatah BM (2015). Relationship of treatment satisfaction to health-related quality of life among Palestinian patients with type 2 diabetes mellitus: findings from a cross-sectional study. J Clin Transl Endocrinol.

[27] Li HB, Wu LJ, Jiang N, Yang PT, Liu SY, Shi XF, Fang YF, Zhao Y, Xu J, Jiang ZY (2020). Treatment satisfaction with rheumatoid arthritis in patients with different disease severity and financial burden: a subgroup analysis of a nationwide survey in China. Chin Med J (Engl).

[28] Abu Al-Fadl EM, Ismail MA, Thabit M, El-Serogy Y (2014). Assessment of health-related quality of life, anxiety and depression in patients with early rheumatoid arthritis. Egypt Rheumatol.

[29] Al-Jabi SW, Seleit DI, Badran A, Koni A, Zyoud SH (2021). Impact of socio-demographic and clinical characteristics on functional disability and health-related quality of life in patients with rheumatoid arthritis: a cross-sectional study from Palestine. Health Qual Life Outcomes.

[30] Giacomelli R, Gorla R, Trotta F, Tirri R, Grassi W, Bazzichi L, Galeazzi M, Matucci-Cerinic M, Scarpa R, Cantini F (2015). Quality of life and unmet needs in patients with inflammatory arthropathies: results from the multicentre, observational RAPSODIA study. Rheumatology (Oxford).

[31] Gong G, Mao J (2016). Health-related quality of life among Chinese patients with rheumatoid arthritis: the predictive roles of fatigue, functional disability, self-efficacy, and social support. Nurs Res.

[32] Franco-Aguirre JQ, Cardona-Tapias AA, Cardona-Arias JA (2017). Health-related quality of life of rheumatic disease patients treated in a specialized IPS in Medellin, **Colombia**. J Med Life.

[33] Gronning K, Rodevand E, Steinsbekk A (2010). Paid work is associated with improved health-related quality of life in patients with rheumatoid arthritis. Clin Rheumatol.

[34] ten Klooster PM, Vonkeman HE, Taal E, Siemons L, Hendriks L, de Jong AJ, Dutmer EA, van Riel PL, van de Laar MA (2013). Performance of the Dutch SF-36 version 2 as a measure of health-related quality of life in patients with rheumatoid arthritis. Health Qual Life Outcomes.

[35] Coons SJ, Alabdulmohsin SA, Draugalis JR, Hays RD (1998). Reliability of an Arabic version of the RAND-36 Health Survey and its equivalence to the US-English version. Med Care.

[36] Alami YZ, Ghanim BT, Zyoud SH (2018). Epworth sleepiness scale in medical residents: quality of sleep and its relationship to quality of life. J Occup Med Toxicol.

[37] Matcham F, Scott IC, Rayner L, Hotopf M, Kingsley GH, Norton S, Scott DL, Steer S (2014). The impact of rheumatoid arthritis on quality-of-life assessed using the SF-36: a systematic review and meta-analysis. Semin Arthritis Rheum.

[38] Liu L, Xu X, Xu N, Wang L (2017). Disease activity, resilience and health-related quality of life in Chinese patients with rheumatoid arthritis: a multi-center, cross-sectional study. Health Qual Life Outcomes.

[39] Zyoud SH, Al-Jabi SW, Sweileh WM, Morisky DE (2013). Relationship of treatment satisfaction to medication adherence: findings from a cross-sectional survey among hypertensive patients in Palestine. Health Qual Life Outcomes.

[40] Atkinson MJ, Sinha A, Hass SL, Colman SS, Kumar RN, Brod M, Rowland CR (2004). Validation of a general measure of treatment satisfaction, the Treatment Satisfaction Questionnaire for Medication (TSQM), using a national panel study of chronic disease. Health Qual Life Outcomes.

[41] Shilbayeh SAR, Alyahya SA, Alshammari NH, Almutairi WA, Shaheen E (2018). Treatment Satisfaction Questionnaire for Medication: Validation of the Translated Arabic Version among Patients Undergoing Warfarin Therapy in Saudi Arabia. Value Health Reg Issues.

[42] Liu L, Xu X, Xu N, Wang L. Disease activity, resilience and health-related quality of life in Chinese patients with rheumatoid arthritis: a multi-center, cross-sectional study. Health Qual Life Outcomes 2017; 15. 10.1186/s12955-017-0725-6 PMC5525274 28738816

[43] Groessl EJ, Ganiats TG, Sarkin AJ (2006). Sociodemographic differences in quality of life in rheumatoid arthritis. Pharmacoeconomics.

[44] Shin SY (2012). The relationship between cognitive and physical function in older adults with rheumatoid arthritis: a literature review. J Gerontol Nurs.

[45] Salaffi F, Carotti M, Gasparini S, Intorcia M, Grassi W (2009). The health-related quality of life in rheumatoid arthritis, ankylosing spondylitis, and psoriatic arthritis: a comparison with a selected sample of healthy people. Health Qual Life Outcomes.

[46] Al-Khatib IA, Arafat RN, Musmar M (2005). Housing environment and women's health in a Palestinian refugee camp. Int J Environ Health Res.

[47] Gurková E, Cáp J, Ziaková K (2009). Quality of life and treatment satisfaction in the context of diabetes self-management education. Int J Nurs Pract.

[48] Sixma HJ, van Campen C, Kerssens JJ, Peters L (2000). Quality of care from the perspective of elderly people: the QUOTE-elderly instrument. Age Ageing.

[49] Krousel-Wood M, Thomas S, Muntner P, Morisky D (2004). Medication adherence: a key factor in achieving blood pressure control and good clinical outcomes in hypertensive patients. Curr Opin Cardiol.

[50] Brekke M, Hjortdahl P, Kvien TK (2001). Involvement and satisfaction: a Norwegian study of health care among 1,024 patients with rheumatoid arthritis and 1,509 patients with chronic noninflammatory musculoskeletal pain. Arthritis Rheum.

[51] Lofland JH, Johnson PT, Ingham MP, Rosemas SC, White JC, Ellis L (2017). Shared decision-making for biologic treatment of autoimmune disease: influence on adherence, persistence, satisfaction, and health care costs. Patient Prefer Adherence.

[52] Lim AY, Ellis C, Brooksby A, Gaffney K (2007). Patient satisfaction with rheumatology practitioner clinics: can we achieve concordance by meeting patients' information needs and encouraging participatory decision making?. Ann Acad Med Singapore.

[53] Chilton F, Collett RA (2008). Treatment choices, preferences and decision-making by patients with rheumatoid arthritis. Musculoskeletal Care.

[54] Wollenhaupt J, Ehlebracht-Koenig I, Groenewegen A, Fricke D (2013). Prioritizing the patient: optimizing therapy in rheumatoid arthritis. Results of a patient questionnaire in northern Germany. Open Access Rheumatol.

[55] Renzi C, Di Pietro C, Tabolli S (2011). Participation, satisfaction and knowledge level of patients with cutaneous psoriasis or psoriatic arthritis. Clin Exp Dermatol.

