# Supplementary material for: Health-related quality of life and treatment satisfaction in Palestinians with rheumatoid arthritis: a cross-sectional study
Source: BMC Rheumatol. 2022 Apr 6;6:19. doi: 10.1186/s41927-022-00251-5 (PMC8985358; doi:10.1186/s41927-022-00251-5)
Supplement: Supplementary file 1 — Additional file 1. Supplemental Table S1-S4: Supplemental Table S1: PCS and MCS subscales with socio-demographic and clinical characteristics; Supplemental Table S2: Mean rank of HRQoL subscales with socio-demographic and clinical characteristics; Supplemental Table S3: PCS and MCS with socio-demographic and clinical characteristics; Supplemental Table S4: Treatment satisfaction with socio-demographic and clinical characteristic. [file 41927_2022_251_MOESM1_ESM.doc]

**Additional file 1: Supplemental Table S1-S4: Supplemental Table S1:** PCS and MCS subscales with socio-demographic and clinical characteristics; **Supplemental Table S2:** Mean rank of HRQoL subscales with socio-demographic and clinical characteristics; **Supplemental Table S3:** PCS and MCS with socio-demographic and clinical characteristics; **Supplemental Table S4:** Treatment satisfaction with socio-demographic and clinical characteristic

**Supplemental Table S1:** PCS and MCS subscales with socio-demographic and clinical characteristics.

|  | **Frequency (%) N =285** | **PF**  **Median**  **[Q1-Q3]** | **RP**  **Median**  **[Q1-Q3]** | **RE**  **Median**  **[Q1-Q3]** | **VT**  **Median**  **[Q1-Q3]** | **MH**  **Median**  **[Q1-Q3]** | **SF**  **Median[Q1-Q3]** | **BP**  **Median[Q1-Q3]** | **GH**  **Median[Q1-Q3]** |
| --- | --- | --- | --- | --- | --- | --- | --- | --- | --- |
| **Hospital*** |  | | | | | | | | |
| Qalqilia | 39(13.7) | 35[30-60] | 0[0-25] | 0[0-100] | 45[25-50] | 56[44-76] | 50[25-75] | 37.5[22.5-47.5] | 35[25-55] |
| Tulkarm | 70(24.6) | 40[35-60] | 0[0-25] | 33.33[0-100] | 40[30-50] | 60[48-72] | 62.5[37.5-75] | 35[22.5-45] | 40[30-50] |
| Jenin | 87(30.5) | 47.5[16.25-60] | 0[0-0] | 0[0-33.33] | 37.5[25-45] | 50[40-74] | 50[25-75] | 33.75[15-46.88] | 32.5[21.25-45] |
| Al Watani | 89(31.2) | 50[25-65] | 0[0-50] | 33.33[0-100] | 45[25-60] | 56[44-72] | 50[25-75] | 45[25-45] | 45[30-50] |
|  | ***pValue*** | *0.791* | *0.705* | *0.210* | *0.597* | *0.884* | *0.417* | *0.332* | *0.593* |
| **Gender**** |  | | | | | | | | |
| Male | 54(18.9) | 50[22.5-67.5] | 0[0-37.5] | 0[0-83.33] | 45[32.5-60] | 64[44-86] | 50[25-75] | 45[22.5-62.5] | 40[22.5-57.5] |
| Female | 231(81.1) | 45[30-60] | 0[0-25] | 0[0-66.67] | 40[25-50] | 56[41-72] | 50[28.13-75] | 35[22.5-45] | 40[25-50] |
|  | ***pValue*** | ***0.777*** | ***0.046*** | ***0.349*** | ***0.169*** | ***0.049*** | ***0.241*** | ***0.266*** | ***0.360*** |
| **Age Group*** |  | | | | | | | | |
| Less than 30 | 21(7.4) | 50[42.5-72.5] | 25[0-62.5] | 0[0-33.33] | 45[27.5-60] | 60[44-76] | 50[43.75-93.75] | 45[33.75-45] | 50[32.5-57.5] |
| 30 years - 39 years | 26(9.1) | 50[36.25-80] | 0[0-18.75] | 50[0-100] | 40[25-45] | 46[41-74] | 50[37.5-75] | 40[25-46.88] | 35[17.5-40] |
| 40 years - 49 years | 69(24.2) | 55[31.25-68.75] | 0[0-18.75] | 0[0-58.33] | 35[25-48.75] | 52[40-72] | 43.75[25-62.5] | 32.5[22.5-45] | 35[16.25-45] |
| 50 years - 59 years | 87(30.5) | 40[23.75-60] | 0[0-6.25] | 0[0-75] | 40[20-51.25] | 54[39-68] | 50[25-75] | 33.75[12.5-45] | 35[23.75-50] |
| ≥60 | 82(28.8) | 40[25-56.25] | 0[0-25] | 33.33[0-100] | 50[28.75-55] | 62[50.25-80] | 56.25[25-78.13] | 45[25-57.5] | 40[30-55] |
|  | ***pValue*** | ***<0.001*** | ***0.043*** | ***0.212*** | ***0.424*** | ***0.059*** | ***0.085*** | ***0.077*** | ***0.183*** |
| **Smoking**** |  | | | | | | | | |
| Smoker | 50(17.5) | 52.5[36.25-75] | 0[0-50] | 16.67[0-100] | 45[35-53.75] | 64[44-80] | 75[28.13-75] | 45[25-46.88] | 45[30-58.75] |
| Non smoker | 235(82.5) | 40[27.5-60] | 0[0-25] | 0[0-66.67] | 40[25-50] | 56[42-72] | 50[25-75] | 35[22.5-45] | 35[25-50] |
|  | ***pValue*** | ***0.396*** | ***0.369*** | ***0.846*** | ***0.784*** | ***0.646*** | ***0.449*** | ***0.909*** | ***0.163*** |
| **Educational Background** |  |  |  |  |  |  |  |  |  |
| below Primary Education | 13(4.6) | 40[13.75-52.5] | 0[0-0] | 0[0-75] | 47.5[17.5-50] | 46[32-58] | 25[6.25-87.5] | 28.75[5.62-43.12] | 32.5[25-62.5] |
| Primary Education | 57(20) | 35[11.25-58.75] | 0[0-25] | 0[0-66.66] | 37.5[20-50] | 48[40-66] | 37.5[15.62-71.87] | 25[13.125-45] | 42.5[21.25-50] |
| Junior High School | 73(25.6) | 50[35-60] | 0[0-25] | 16.66[0-100] | 40[25-50] | 60[44-83] | 50[37.5-84.37] | 35[22.5-45] | 40[26.25-53.75] |
| Senior high School | 57(20) | 50[30-70] | 0[0-50] | 0[0-33.33] | 45[30-55] | 56[40-68] | 50[37.5-75] | 45[32.5-57.5] | 30[20-45] |
| Collage or more | 85(29.8) | 45[35-60] | 0[0-25] | 33.33[0-66.66] | 40[25-50] | 68[48-76] | 50[37.5-75] | 45[32.5-47.5] | 40[30-50] |
|  | ***pValue*** | **0.009** | **0.020** | 0.450 | 0.489 | 0.188 | **0.016** | **< 0.001** | 0.851 |
| **Marital status** |  |  |  |  |  |  |  |  |  |
| Single | 53(18.6) | 50[25-60] | 0[0-25] | 0[0-66.66] | 40[20-55] | 52[40-72] | 50[25-75] | 45[22.5-45] | 45[25-50] |
| Married | 199(69.8) | 45[30-60] | 0[0-25] | 0[0-100] | 40[28.75-50] | 60[44-73] | 50[25-75] | 35[22.5-45] | 35[25-50] |
| Divorced/ Widowed | 33(11.6) | 42.5[25-72.5] | 0[0-50] | 33.33[0-100] | 45[16.25-53.75] | 58[41-66] | 62.5[37.5-84.37] | 46.25[25-65] | 40[26.25-50] |
|  | ***pValue*** | 0.317 | 0.946 | 0.512 | 0.643 | 0.139 | 0.531 | 0.658 | 0.446 |
| **Employment** |  |  |  |  |  |  |  |  |  |
| Employed | 67(23.5) | 55[42.5-75] | 0[0-25] | 33.33[0-100] | 40[25-56.25] | 54[40-73] | 50[25-75] | 45[32.5-59.37] | 45[28.75-56.25] |
| unemployed | 199(69.8) | 40[30-60] | 0[0-25] | 0[0-66.66] | 40[25-50] | 60[44-72] | 50[31.25-75] | 35[22.5-45] | 35[25-50] |
| stopped because of RA | 19(6.7) | 30[13.75-47.5] | 0[0-0] | 0[0-8.33] | 32.5[22.5-45] | 58[38-63] | 50[18.75-65.62] | 31.25[20-57.5] | 20[10-37.5] |
|  | ***pValue*** | **< 0.001** | **0.013** | 0.094 | 0.207 | 0.449 | 0.275 | 0.051 | **0.015** |
| **Place of residence** |  |  |  |  |  |  |  |  |  |
| City | 101(35.4) | 45[30-60] | 0[0-50] | 0[0-66.66] | 45[25-50] | 60[44-76] | 50[31.25-75] | 35[22.5-52.5] | 45[25-55] |
| Village | 169(59.3) | 45[30-60] | 0[0-0] | 0[0-91.66] | 40[25-53.75] | 56[41-72] | 50[25-75] | 35[22.5-45] | 35[25-45] |
| Refugee Camp | 15(5.3) | 40[3.75-76.25] | 0[0-75] | 0[0-75] | 37.5[7.5-56.25] | 52[41-84] | 37.5[0-75] | 27.5[2.5-86.25] | 25[12.5-52.5] |
|  | ***pValue*** | 0.688 | **0.046** | 0.709 | 0.470 | 0.799 | 0.985 | 0.144 | 0.076 |

|  | **Frequency (%) N =285** | **PF**  **Median**  **[Q1-Q3]** | **RP**  **Median**  **[Q1-Q3]** | **RE**  **Median**  **[Q1-Q3]** | **VT**  **Median**  **[Q1-Q3]** | **MH**  **Median**  **[Q1-Q3]** | **SF Median**  **[Q1-Q3]** | **BP Median**  **[Q1-Q3]** | **GH Median**  **[Q1-Q3]** |
| --- | --- | --- | --- | --- | --- | --- | --- | --- | --- |
| **Household Income*** |  | | | | | | | | |
| Low: Less than 400 JD | 145(50.9) | 40[25-55] | 0[0-0] | 0[0-66.67] | 35[20-50] | 52[40-68] | 50[25-75] | 32.5[22.5-45] | 35[20-45] |
| Moderate: Between 400-1000 JD | 119(41.8) | 50[35-71.25] | 0[0-50] | 33.33[0-100] | 45[30-56.25] | 64[47.25-80] | 62.5[37.5-75] | 45[35-57.5] | 45[30-50] |
| High: More than 1000 JD | 20(7) | 55[40-85] | 12.5[0-62.5] | 16.67[0-58.33] | 45[41.25-48.75] | 74[59-86] | 62.5[28.13-96.88] | 38.75[25-60] | 37.5[31.25-51.25] |
|  | ***pValue*** | ***<0.001*** | ***0.003*** | ***0.447*** | ***0.038*** | ***0.014*** | ***0.084*** | ***0.012*** | ***0.002*** |
| **BMI category*** |  | | | | | | | | |
| Underweight / Normal | 52(18.2) | 50[31.25-60] | 0[0-25] | 16.67[0-100] | 40[27.5-57.5] | 60[41-87] | 50[37.5-84.38] | 45[33.13-56.25] | 40[25-60] |
| Overweight | 129(45.3) | 45[35-64.03] | 0[0-25] | 16.67[0-100] | 45[25-55] | 60[44-76] | 50[25-75] | 35[22.5-46.88] | 45[30-53.75] |
| Obese | 104(36.5) | 40[25-60] | 0[0-0] | 0[0-50] | 35[25-50] | 52[40-68] | 50[31.25-75] | 35[22.5-45] | 35[25-45] |
|  | ***pValue*** | ***0.191*** | ***0.075*** | ***0.286*** | ***0.157*** | ***0.204*** | ***0.461*** | ***0.151*** | ***0.023*** |
| **Disease Activity*** |  | | | | | | | | |
| Inactive | 124(43.5) | 55[40-75] | 0[0-75] | 33.33[0-100] | 45[35-55] | 60[44-78] | 50[37.5-75] | 45[32.5-61.25] | 50[35-60] |
| Low to moderate | 98(34.4) | 50[35-60] | 0[0-0] | 0[0-66.67] | 40[25-50] | 56[44-72] | 50[37.5-84.38] | 35[32.5-45] | 35[25-45] |
| High | 61(21.4) | 22.5[15-35] | 0[0-0] | 0[0-25] | 25[15-38.75] | 52[37-68] | 25[3.13-62.5] | 22.5[2.5-35] | 25[10-42.5] |
|  | ***pValue*** | ***<0.001*** | ***<0.001*** | ***<0.001*** | ***<0.001*** | ***0.001*** | ***0.002*** | ***<0.001*** | ***<0.001*** |
| **Treatment Status**** |  | | | | | | | | |
| Regular treatment | 239(83.9) | 45[30-60] | 0[0-0] | 0[0-66.67] | 40[25-50] | 56[44-72] | 50[37.5-75] | 35[22.5-45] | 40[25-50] |
| non formal treatment | 46(16.1) | 40[20-60.28] | 12.5[0-56.25] | 0[0-100] | 30[18.75-55] | 58[35-77] | 43.75[21.88-75] | 32.5[20-50] | 37.5[15-51.25] |
|  | ***pValue*** | ***0.220*** | ***0.262*** | ***0.282*** | ***0.385*** | ***0.943*** | ***0.624*** | ***0.899*** | ***0.213*** |

|  | **Frequency (%) N =285** | **PF**  **Median**  **[Q1-Q3]** | | **RP**  **Median**  **[Q1-Q3]** | | **RE**  **Median**  **[Q1-Q3]** | | | **VT**  **Median**  **[Q1-Q3]** | | **MH**  **Median**  **[Q1-Q3]** | | **SF**  **Median[Q1-Q3]** | | **BP**  **Median[Q1-Q3]** | | **GH**  **Median**  **[Q1-Q3]** |
| --- | --- | --- | --- | --- | --- | --- | --- | --- | --- | --- | --- | --- | --- | --- | --- | --- | --- |
| **Duration of disease (years)*** |  | | | | | | | | | | | | | | | | |
|  | 9(3.2) | | 42.5[28.75-70] | | 0[0-31.25] | | 50[0-66.67] | 45[22.5-65] | | 50[21-68] | | 31.25[25-65.63] | | 43.75[20-59.38] | | 47.5[28.75-50] | |
| 1-3 years | 73(25.6) | | 50[31.25-73.75] | | 0[0-68.75] | | 33.33[0-100] | 40[25-55] | | 56[44-71] | | 50[28.13-75] | | 45[33.13-57.5] | | 42.5[35-57.5] | |
| 4-5 years | 52(18.2) | | 45[23.75-60] | | 0[0-56.25] | | 0[0-75] | 45[25-51.25] | | 54[44-69] | | 50[37.5-90.63] | | 35[22.5-45.63] | | 42.5[23.75-55] | |
| ≥ 5 years | 150(52.6) | | 40[25-57.5] | | 0[0-0] | | 0[0-66.67] | 40[25-50] | | 60[42-76] | | 50[25-75] | | 35[22.5-45] | | 35[25-45] | |
|  | ***pValue*** | | ***0.064*** | | ***0.007*** | | ***0.445*** | ***0.560*** | | ***0.595*** | | ***0.088*** | | ***0.162*** | | ***0.026*** | |
| **Total number of Comorbid diseases*** |  | | | | | | | | | | | | | | | | |
| zero | 91(31.9) | | 55[30-80] | | 0[0-50] | | 33.33[0-100] | 45[30-60] | | 60[44-84] | | 50[37.5-87.5] | | 45[25-47.5] | | 40[25-55] | |
| One Comorbid Disease | 69(24.2) | | 50[35-60] | | 0[0-75] | | 33.33[0-100] | 40[30-50] | | 52[44-68] | | 50[25-75] | | 35[25-60] | | 45[40-55] | |
| two Comorbid Disease | 53(18.6) | | 50[30-60] | | 0[0-0] | | 0[0-100] | 45[20-50] | | 60[48-68] | | 50[25-62.5] | | 32.5[22.5-45] | | 35[25-45] | |
| Three Comorbid Disease | 37(13) | | 40[30-55] | | 0[0-12.5] | | 0[0-66.67] | 30[22.5-42.5] | | 60[42-74] | | 50[37.5-68.75] | | 35[22.5-40] | | 30[17.5-42.5] | |
| ≥ 4 Comorbid Disease | 35(12.3) | | 35[15-45] | | 0[0-0] | | 0[0-33.33] | 30[15-50] | | 48[32-72] | | 62.5[12.5-75] | | 35[12.5-45] | | 35[20-45] | |
|  | ***pValue*** | | ***<0.001*** | | ***<0.001*** | | ***0.015*** | ***0.007*** | | ***0.073*** | | ***0.004*** | | ***<0.001*** | | ***0.003*** | |
| **Total number of medication*** |  | | | | | | | | | | | | | | | | |
| 1-3 medications | 41(14.4) | | 60[40-90] | | 0[0-100] | | 33.33[0-100] | 40[30-70] | | 56[40-88] | | 50[37.5-87.5] | | 45[32.5-47.5] | | 50[25-55] | |
| 4-6 medications | 122(42.8) | | 50[35-60] | | 0[0-25] | | 0[0-66.67] | 45[25-53.75] | | 52[44-68] | | 50[25-75] | | 45[24.38-45] | | 40[26.25-53.75] | |
| ≥7 | 122(42.8) | | 35[20-55] | | 0[0-0] | | 0[0-100] | 35[25-50] | | 60[40-76] | | 50[25-75] | | 32.5[22.5-45] | | 35[20-45] | |
|  | ***pValue*** | | ***<0.001*** | | ***0.001*** | | ***0.267*** | ***0.104*** | | ***0.346*** | | ***0.279*** | | ***0.010*** | | ***0.021*** | |

***** The Kruskal-Wallis test was used
****** The Mann-Whitney test was used

**Supplemental Table S2: Mean rank of HRQoL subscales with socio-demographic and clinical characteristics**

|  | | **Frequency (%) N =285** | **PF**  **Mean Rank** | **RP**  **Mean Rank** | **BP**  **Mean Rank** | **GH**  **Mean Rank** | **RE**  **Mean Rank** | **VT**  **Mean Rank** | **MH**  **Mean Rank** | **SF**  **Mean Rank** |
| --- | --- | --- | --- | --- | --- | --- | --- | --- | --- | --- |
| **Hospital** |  | | | | | | | | | |
| Qalqilia | | 39(13.7) | 153.65 | 155.64 | 158.60 | 156.87 | 153.04 | 144.33 | 149.28 | 152.27 |
| Tulkarm | | 70(24.6) | 138.21 | 142.77 | 132.13 | 139.37 | 143.54 | 141.52 | 144.93 | 148.46 |
| Jenin | | 87(30.5) | 140.15 | 140.22 | 138.44 | 136.59 | 129.36 | 134.78 | 137.59 | 131.04 |
| Al Watani | | 89(31.2) | 144.88 | 140.35 | 149.17 | 146.04 | 151.51 | 151.62 | 144.02 | 146.34 |
| **Gender** | |  | | | | | | | | |
| Male | | 54(18.9) | 145.85 | 160.92 | 154.19 | 152.20 | 151.79 | 156.84 | 162.81 | 131.30 |
| Female | | 231(81.1) | 142.33 | 138.81 | 140.39 | 140.85 | 140.95 | 139.76 | 138.37 | 145.74 |
| **Age Group** | |  | | | | | | | | |
| Less than 30 | | 21(7.4) | 203.74 | 186.02 | 180.60 | 176.69 | 146.90 | 162.76 | 153.79 | 186.98 |
| 30 years - 39 years | | 26(9.1) | 166.62 | 147.60 | 139.08 | 131.50 | 157.10 | 135.48 | 133.62 | 157.73 |
| 40 years - 49 years | | 69(24.2) | 162.10 | 147.78 | 140.80 | 133.14 | 126.62 | 135.11 | 124.97 | 134.37 |
| 50 years - 59 years | | 87(30.5) | 131.80 | 132.61 | 128.14 | 138.11 | 140.57 | 136.91 | 138.66 | 139.32 |
| ≥60 | | 82(28.8) | 115.76 | 137.52 | 152.23 | 151.50 | 153.89 | 153.43 | 162.99 | 138.24 |
| **Smoking** | |  | | | | | | | | |
| Smoker | | 50(17.5) | 151.96 | 134.54 | 141.80 | 157.72 | 141.09 | 140.11 | 147.86 | 135.08 |
| Non smoker | | 235(82.5) | 141.09 | 144.80 | 143.26 | 139.87 | 143.41 | 143.61 | 141.97 | 144.69 |
| **Educational Background** | |  | | | | | | | | |
| below Primary Education | | 13(4.6) | 103.15 | 122.88 | 122.46 | 130.69 | 122.04 | 130.04 | 110.69 | 100.54 |
| Primary Education | | 57(20) | 118.54 | 135.35 | 119.22 | 141.75 | 141.25 | 134.88 | 131.49 | 127.49 |
| Junior High School | | 73(25.6) | 138.84 | 125.12 | 120.88 | 138.20 | 133.69 | 134.99 | 142.38 | 132.13 |
| Senior high School | | 57(20) | 162.81 | 162.00 | 167.90 | 141.46 | 146.25 | 156.23 | 139.84 | 160.82 |
| Collage or more | | 85(29.8) | 155.79 | 153.82 | 164.38 | 150.88 | 153.20 | 148.44 | 158.31 | 157.28 |
|  | | **Frequency (%) N =285** | **PF**  **Mean Rank** | **RP**  **Mean Rank** | **BP**  **Mean Rank** | **GH**  **Mean Rank** | **RE**  **Mean Rank** | **VT**  **Mean Rank** | **MH**  **Mean Rank** | **SF**  **Mean Rank** |
| **Marital status** | |  | | | | | | | | |
| Single | | 53(18.6) | 150.81 | 145.96 | 149.68 | 150.64 | 151.58 | 151.99 | 163.25 | 151.76 |
| Married | | 199(69.8) | 144.09 | 142.18 | 140.08 | 143.51 | 139.56 | 141.65 | 138.43 | 142.56 |
| Divorced/ Widowed | | 33(11.6) | 123.88 | 143.17 | 149.89 | 127.68 | 149.97 | 136.70 | 138.03 | 131.59 |
| **Employment** | |  | | | | | | | | |
| Employed | | 67(23.5) | 170.40 | 159.46 | 161.22 | 162.61 | 158.07 | 148.21 | 151.49 | 147.03 |
| unemployed | | 199(69.8) | 138.78 | 141.11 | 139.58 | 140.13 | 140.22 | 144.28 | 141.80 | 144.39 |
| stopped because of RA | | 19(6.7) | 90.58 | 104.74 | 114.58 | 103.89 | 118.97 | 111.26 | 125.63 | 114.26 |
| **Place of residence** | |  | | | | | | | | |
| City | | 101(35.4) | 148.66 | 157.48 | 155.51 | 155.05 | 148.06 | 140.04 | 147.30 | 141.88 |
| Village | | 169(59.3) | 140.05 | 134.64 | 137.00 | 138.79 | 140.35 | 146.68 | 140.90 | 143.59 |
| Refugee Camp | | 15(5.3) | 138.07 | 139.67 | 126.40 | 109.27 | 138.83 | 121.40 | 137.73 | 143.90 |
| **Household Income** | |  | | | | | | | | |
| Low: Less than 400 JD | | 145(50.9) | 125.69 | 133.22 | 128.44 | 127.21 | 138.40 | 130.93 | 128.64 | 132.02 |
| Moderate: Between 400-1000 JD | | 119(41.8) | 154.57 | 145.55 | 156.15 | 153.97 | 144.47 | 152.28 | 156.41 | 152.91 |
| High: More than 1000 JD | | 20(7) | 192.60 | 191.65 | 163.18 | 185.15 | 160.53 | 168.15 | 160.23 | 156.53 |
| **BMI category** | |  | | | | | | | | |
| Underweight / Normal | | 52(18.2) | 154.98 | 151.08 | 162.36 | 155.60 | 154.74 | 149.81 | 155.69 | 151.99 |
| Overweight | | 129(45.3) | 147.10 | 150.26 | 141.06 | 152.09 | 144.91 | 150.21 | 146.41 | 145.22 |
| Obese | | 104(36.5) | 131.92 | 129.96 | 135.73 | 125.43 | 134.75 | 130.66 | 132.42 | 135.75 |
| **Disease Activity** | |  | | | | | | | | |
| Inactive | | 124(43.5) | 168.02 | 162.46 | 174.39 | 174.49 | 164.39 | 169.28 | 161.10 | 159.81 |
| Low to moderate | | 98(34.4) | 137.64 | 133.51 | 128.86 | 121.56 | 128.07 | 135.73 | 121.39 | 134.63 |
| High | | 61(21.4) | 96.11 | 114.06 | 97.26 | 108.80 | 118.86 | 96.61 | 136.28 | 117.64 |
| **Treatment Status** | |  | | | | | | | | |
| Regular treatment | | 239(83.9) | 140.38 | 140.86 | 143.27 | 145.66 | 140.86 | 144.86 | 143.15 | 144.04 |
| non formal treatment | | 46(16.1) | 156.63 | 154.10 | 141.60 | 129.20 | 154.14 | 133.36 | 142.21 | 137.60 |
| **Duration of disease (years)** | |  | | | | | | | | |
| <1 | | 9(3.2) | 128.83 | 132.50 | 149.39 | 152.28 | 135.61 | 143.89 | 114.94 | 99.44 |
| 1-3 years | | 73(25.6) | 160.97 | 159.92 | 160.68 | 166.37 | 153.14 | 146.20 | 145.32 | 144.68 |
| 4-5 years | | 52(18.2) | 150.96 | 158.77 | 137.19 | 139.98 | 146.85 | 154.39 | 134.00 | 162.87 |
| >5 years | | 150(52.6) | 131.40 | 128.98 | 135.08 | 131.17 | 136.23 | 136.49 | 145.73 | 136.96 |
| **Total number of Comorbid diseases** | |  | | | | | | | | |
| zero | | 91(31.9) | 180.02 | 162.30 | 164.25 | 153.46 | 153.65 | 164.15 | 155.84 | 167.62 |
| One Comorbid Disease | | 69(24.2) | 144.56 | 158.33 | 155.08 | 162.70 | 158.79 | 147.93 | 136.13 | 136.03 |
| two Comorbid Disease | | 53(18.6) | 142.19 | 133.22 | 136.52 | 141.15 | 135.15 | 134.83 | 155.90 | 141.06 |
| Three Comorbid Disease | | 37(13) | 114.43 | 120.45 | 112.03 | 117.86 | 127.55 | 123.04 | 131.51 | 128.00 |
| ≥ 4 Comormid Disease | | 35(12.3) | 75.10 | 101.26 | 106.50 | 106.33 | 112.39 | 111.77 | 115.77 | 111.54 |
| **Total number of medication** | |  | | | | | | | | |
| 1-3 medications | | 41(14.4) | 179.32 | 168.91 | 171.48 | 164.77 | 160.66 | 163.80 | 159.17 | 153.38 |
| 4-6 medications | | 122(42.8) | 161.03 | 152.18 | 147.83 | 150.27 | 141.58 | 145.83 | 137.53 | 148.21 |
| ≥7 | | 122(42.8) | 112.76 | 125.11 | 128.60 | 128.42 | 138.48 | 133.18 | 143.03 | 134.30 |

**Supplemental Table S3: PCS and MCS with socio-demographic and clinical characteristics**

|  | **Frequency (%) N =285** | **PCS** | | **MCS** | |
| --- | --- | --- | --- | --- | --- |
|  |  | **Median[Q1-Q3]** | **Mean Rank** | **Median[Q1-Q3]** | **Mean Rank** |
| **Hospital** | | | | | |
| Qalqilia | 39(13.7) | 30[18.13-45.63] | 160.04 | 39.38[31.5-68] | 152.69 |
| Tulkarm | 70(24.6) | 33.13[23.4-41.88] | 137.11 | 42.75[35.33-69.17] | 144.25 |
| Jenin | 87(30.5) | 27.19[12.97-44.38] | 136.99 | 36.79[22-59.77] | 128.93 |
| Al Watani | 89(31.2) | 35[26.25-51.25] | 146.04 | 50.04[29.75-67.5] | 151.53 |
|  | ***pValue*** | ***0.456*** | | ***0.257*** | |
| **Gender** | | | | | |
| Male | 54(18.9) | 35[19.38-52.5] | 156.41 | 41[29.38-71.79] | 150.34 |
| Female | 231(81.1) | 30.31[23.13-41.88] | 139.87 | 40.96[26.69-66.81] | 141.28 |
|  | ***pValue*** | ***0.184*** | | ***0.467*** | |
| **Age Group** |  | | | | |
| Less than 30 | 21(7.4) | 41.25[35.94-57.5] | 202.24 | 38.75[32.92-62.65] | 166.17 |
| 30 years - 39 years | 26(9.1) | 31.88[22.19-44.84] | 149.67 | 40.08[32.25-65.76] | 153.00 |
| 40 years - 49 years | 69(24.2) | 29.69[19.69-43.75] | 147.18 | 35.81[25.78-56.06] | 126.20 |
| 50 years - 59 years | 87(30.5) | 29.06[16.72-40.47] | 129.16 | 42.46[19.78-59.58] | 138.14 |
| ≥60 | 82(28.8) | 32.5[24.06-46.72] | 136.88 | 53.52[35.69-69.47] | 153.19 |
|  |  | ***0.007*** | | ***0.167*** | |
| **Smoking** | | | | | |
| Smoker | 50(17.5) | 33.75[26.41-53.44] | 143.93 | 51.13[32.16-69.64] | 138.74 |
| Non smoker | 235(82.5) | 30.63[21.25-42.5] | 142.80 | 40.5[26.5-62.23] | 143.91 |
|  | ***pValue*** | ***0.930*** | | ***0.687*** | |
| **Educational Background** | | | | | |
| below Primary Education | 13(4.6) | 24.38[11.56-39.53] | 112.31 | 35.25[16.69-53.94] | 109.65 |
| Primary Education | 57(20) | 28.13[11.56-42.34] | 122.48 | 31.94[20.71-64.86] | 131.42 |
| Junior High School | 73(25.6) | 30.63[21.09-47.66] | 127.27 | 47.6[26.78-75.22] | 132.19 |
| Senior high School | 57(20) | 30[25-52.5] | 163.38 | 41[33.38-59.08] | 153.17 |
| Collage or more | 85(29.8) | 35[26.09-42.66] | 161.29 | 48.13[36.78-63.53] | 158.33 |
|  | ***pValue*** | ***0.003*** | | ***0.081*** | |
| **Marital status** | | | | | |
| Single | 53(18.6) | 35[14.38-41.88] | 150.08 | 37.13[23.13-55.25] | 154.52 |
| Married | 199(69.8) | 30[22.34-43.13] | 142.37 | 41.77[28.19-66.69] | 140.56 |
| Divorced/ Widowed | 33(11.6) | 35.94[23.75-53.91] | 135.41 | 58.65[22.31-69.13] | 139.20 |
|  | ***pValue*** | ***0.711*** | | ***0.527*** | |
| **Employment** | | | | | |
| Employed | 67(23.5) | 38.75[28.44-51.25] | 167.54 | 41.58[31.84-67.88] | 153.45 |
| unemployed | 199(69.8) | 30[22.81-40.63] | 140.00 | 42.33[27.69-67.33] | 142.49 |
| stopped because of RA | 19(6.7) | 23.75[11.72-32.5] | 87.89 | 33.5[19.69-49.96] | 111.47 |
|  | ***pValue*** | ***0.001*** | | ***0.145*** | |
| **Place of residence** | | | | | |
| City | 101(35.4) | 35.63[25.31-51.56] | 158.08 | 44[28.81-67.92] | 146.07 |
| Village | 169(59.3) | 30[22.03-40] | 135.60 | 40.94[27.28-61.5] | 141.88 |
| Refugee Camp | 15(5.3) | 21.88[5.94-72.5] | 124.83 | 31.75[13.13-71.56] | 134.90 |
|  | ***pValue*** | ***0.065*** | | ***0.854*** | |
| **Household Income** | | | | | |
| Low: Less than 400 JD | 145(50.9) | 27.5[17.5-40] | 124.04 | 35.88[22.75-54.5] | 130.33 |
| Moderate: Between 400-1000 JD | 119(41.8) | 35.94[27.19-52.81] | 156.42 | 53.85[38.81-70.56] | 154.06 |
| High: More than 1000 JD | 20(7) | 37.81[24.69-62.19] | 193.55 | 51.17[33.66-69.3] | 161.93 |
|  | ***pValue*** | ***<0.001*** | | ***0.036*** | |
| **BMI category** | | | | | |
| Underweight / Normal | 52(18.2) | 35.31[29.06-44.84] | 160.09 | 46.29[32-70.88] | 156.98 |
| Overweight | 129(45.3) | 33.13[23.13-51.09] | 148.91 | 47.5[33.66-67.96] | 146.73 |
| Obese | 104(36.5) | 27.5[19.69-39.38] | 127.13 | 37.13[23.63-56.35] | 131.38 |
|  | ***pValue*** | ***0.034*** | | ***0.148*** | |
|  | ***Frequency (%) N =285*** | ***PCS*** | | ***MCS*** | |
| **Disease Activity** | | | | | |
| Inactive | 124(43.5) | 38.75[30.31-55.31] | 175.65 | 55.25[36.73-70.77] | 169.50 |
| Low to moderate | 98(34.4) | 30.31[25.63-40] | 130.14 | 42.25[28.91-65.76] | 127.00 |
| High | 61(21.4) | 17.81[8.91-29.22] | 92.66 | 26.81[16.59-44.06] | 110.20 |
|  | ***pValue*** | ***<0.001*** | | ***<0.001*** | |
| **Treatment Status** | | | | | |
| Regular treatment | 239(83.9) | 31.25[23.13-42.5] | 141.49 | 42.17[28.5-66.63] | 142.93 |
| non formal treatment | 46(16.1) | 32.5[13.75-52.19] | 150.86 | 38.69[19.34-69.25] | 143.37 |
|  | ***pValue*** | ***0.480*** | | ***0.974*** | |
| **Duration of disease (years)** | | | | | |
| <1 | 9(3.2) | 35.31[20.47-50] | 131.67 | 39.69[27.44-64.14] | 119.33 |
| 1-3 years | 73(25.6) | 35.31[27.03-60.63] | 164.42 | 47.5[31.81-67.63] | 149.38 |
| 4-5 years | 52(18.2) | 35.31[24.84-52.97] | 152.21 | 43.88[26.81-62.25] | 151.34 |
| >5 years | 150(52.6) | 30[20.31-36.25] | 129.12 | 39.38[25.94-64.31] | 137.48 |
|  | ***pValue*** | ***0.018*** | | ***0.495*** | |
| **Total number of Comorbid diseases** | | | | | |
| zero | 91(31.9) | 36.25[23.75-52.5] | 171.10 | 55.25[30.5-69.79] | 163.27 |
| One Comorbid Disease | 69(24.2) | 35[28.75-54.38] | 158.63 | 42.75[27.25-67.67] | 147.62 |
| two Comorbid Disease | 53(18.6) | 30.63[23.13-42.5] | 138.42 | 41.38[31.75-54.5] | 140.96 |
| Three Comorbid Disease | 37(13) | 26.88[21.56-33.75] | 107.38 | 39.38[26.75-55.98] | 125.47 |
| ≥ 4 Comorbid Disease | 35(12.3) | 26.25[10-37.5] | 83.70 | 37.13[15.5-57.71] | 102.80 |
|  | ***pValue*** | ***0.501*** | | ***<0.001*** | |
| **Total number of medication** | | | | | |
| 1-3 medications | 41(14.4) | 38.75[27.5-76.88] | 178.95 | 39.38[31.5-81.75] | 162.94 |
| 4-6 medications | 122(42.8) | 34.38[25.63-50.63] | 156.30 | 41.88[26.97-62.32] | 143.20 |
| ≥7 | 122(42.8) | 28.13[19.38-35.63] | 117.61 | 40.75[26.19-66.84] | 136.10 |
|  | ***pValue*** | ***<0.001*** | | ***0.196*** | |

**Supplemental Table S4: Treatment satisfaction with socio-demographic and clinical characteristic**

|  |  |  |  |  |  |  | |  |  |  |  |
| --- | --- | --- | --- | --- | --- | --- | --- | --- | --- | --- | --- |
|  | **Frequency (%) N =285** | Effectiveness | | Side Effects | |  | Convenience | | | Overall satisfaction | |
|  |  | Median[Q1-Q3] | Mean Rank | Median[Q1-Q3] | Mean Rank | Median[Q1-Q3] | |  | Mean Rank | Median[Q1-Q3] | Mean Rank |
| **Hospital** |  | | | | | | | | | | |
| Qalqilia | 39(13.7) | 61.11[44.44-66.67] | 145.78 | 50[31.25-62.5] | 66.77 | 55.56[50-61.11] | |  | 145.51 | 45.83[29.17-61.11] | 143.67 |
| Tulkarm | 70(24.6) | 55.56[50-77.78] | 140.73 | 50[37.5-68.75] | 76.75 | 55.56[50-66.67] | |  | 141.99 | 52.78[37.5-69.44] | 141.53 |
| Jenin | 87(30.5) | 55.56[50-70.83] | 151.10 | 40.63[18.75-65.63] | 64.76 | 50[44.44-66.67] | |  | 142.44 | 53.47[36.46-69.1] | 150.63 |
| Al Watani | 89(31.2) | 55.56[50-66.67] | 135.65 | 50[25-62.5] | 70.44 | 61.11[50-66.67] | |  | 143.24 | 52.78[37.5-69.44] | 136.40 |
|  | ***pValue*** | ***0.643*** | | ***0.611*** | |  | ***0.997*** | | | ***0.718*** | |
| **Gender** |  |  |  |  |  |  | |  |  |  |  |
| Male | 54(18.9) | 61.11[50-72.22] | 141.31 | 50[40.63-75] | 81.02 | 55.56[50-66.67] | |  | 144.03 | 54.17[32.64-76.39] | 140.06 |
| Female | 231(81.1) | 55.56[50-66.67] | 143.40 | 43.75[25-62.5] | 67.58 | 55.56[44.44-66.67] | |  | 142.76 | 52.78[37.5-68.06] | 143.69 |
|  | ***pValue*** | ***0.866*** | | ***0.130*** | |  | ***0.918*** | | | ***0.770*** | |
| **Age Group** |  |  |  |  |  |  | |  |  |  |  |
| Less than 30 | 21(7.4) | 61.11[50-66.67] | 136.02 | 43.75[34.38-56.25] | 70.33 | 61.11[41.67-66.67] | |  | 129.86 | 45.83[32.64-69.44] | 140.40 |
| 30 years - 39 years | 26(9.1) | 58.33[50-70.83] | 148.92 | 53.13[39.06-81.25] | 83.04 | 58.34[45.83-66.67] | |  | 141.44 | 61.81[31.25-69.44] | 143.40 |
| 40 years - 49 years | 69(24.2) | 55.56[34.72-72.22] | 133.79 | 37.5[18.75-62.5] | 62.08 | 55.56[44.44-66.67] | |  | 148.93 | 49.31[29.51-62.5] | 136.88 |
| 50 years - 59 years | 87(30.5) | 55.56[50-66.67] | 150.03 | 50[25-62.5] | 66.15 | 55.56[43.05-61.11] | |  | 136.61 | 53.47[37.15-69.44] | 146.95 |
| ≥60 | 82(28.8) | 61.11[50-66.67] | 143.20 | 50[31.25-75] | 76.13 | 61.11[50-66.67] | |  | 148.65 | 48.61[42.71-69.44] | 144.49 |
|  |  | ***0.771*** | | ***0.414*** | |  | ***0.762*** | | | ***0.959*** | |
| **Smoking** |  |  |  |  |  |  | |  |  |  |  |
| Smoker | 50(17.5) | 63.89[45.83-72.22] | 141.30 | 56.25[40.63-62.5] | 81.08 | 55.56[45.83-59.72] | |  | 136.14 | 52.78[31.25-74.31] | 146.15 |
| Non smoker | 235(82.5) | 55.56[50-66.67] | 143.36 | 43.75[25-62.5] | 67.57 | 61.11[50-66.67] | |  | 144.46 | 52.78[37.5-68.75] | 142.33 |
|  | ***pValue*** | ***0.871*** | | ***0.128*** | |  | ***0.511*** | | | ***0.765*** | |
| **Educational Background** |  |  |  |  |  |  | |  |  |  |  |
| below Primary Education | 13(4.6) | 63.89[52.78-79.17] | 171.77 | 53.13[37.5-82.81] | 81.06 | 55.56[44.44-66.67] | |  | 142.42 | 45.83[45.83-61.11] | 148.92 |
| Primary Education | 57(20) | 55.56[50-66.67] | 139.35 | 31.25[14.06-62.5] | 56.59 | 55.56[38.89-61.11] | |  | 128.17 | 45.83[37.5-66.67] | 149.83 |
| Junior High School | 73(25.6) | 55.56[40.28-72.22] | 136.12 | 50[18.75-67.19] | 69.27 | 55.56[50-65.28] | |  | 128.89 | 58.33[32.29-69.44] | 135.31 |
| Senior high School | 57(20) | 55.56[50-66.67] | 146.84 | 50[37.5-62.5] | 74.59 | 55.56[50-66.67] | |  | 153.27 | 45.83[29.17-61.11] | 141.98 |
| Collage or more | 85(29.8) | 55.56[50-66.67] | 144.38 | 50[35.94-64.06] | 74.65 | 61.11[50-66.67] | |  | 158.26 | 56.94[37.5-69.44] | 144.80 |
|  | ***pValue*** | ***0.665*** | | ***0.310*** | |  | ***0.092*** | | | ***0.886*** | |

| **Marital status** |  |  |  |  |  |  |  |  |  |
| --- | --- | --- | --- | --- | --- | --- | --- | --- | --- |
| Single | 53(18.6) | 55.56[44.44-61.11] | 134.06 | 43.75[31.25-56.25] | 63.22 | 55.56[38.89-61.11] | 133.11 | 45.83[29.17-61.11] | 127.42 |
| Married | 199(69.8) | 55.56[50-72.22] | 145.74 | 50[25-64.06] | 72.79 | 55.56[50-66.67] | 144.73 | 53.47[37.5-69.44] | 146.99 |
| Divorced/ Widowed | 33(11.6) | 61.11[50-70.83] | 140.85 | 37.5[25-67.19] | 64.72 | 61.11[51.39-66.67] | 148.42 | 50[30.9-69.1] | 143.97 |
|  | ***pValue*** | ***0.644*** | | ***0.470*** | | ***0.600*** | | ***0.305*** | |
| **Employment** |  |  |  |  |  |  |  |  |  |
| Employed | 67(23.5) | 55.56[43.06-68.06] | 144.17 | 50[31.25-64.06] | 76.84 | 55.56[48.61-62.5] | 148.09 | 52.78[37.15-68.4] | 143.41 |
| unemployed | 199(69.8) | 55.56[50-66.67] | 144.20 | 50[28.13-62.5] | 69.21 | 55.56[47.22-66.67] | 141.95 | 52.78[37.5-69.44] | 144.49 |
| stopped because of RA | 19(6.7) | 58.33[50-62.5] | 126.26 | 31.25[4.69-65.63] | 53.50 | 52.78[48.61-66.67] | 136.00 | 40.97[30.21-60.07] | 125.92 |
|  | ***pValue*** | ***0.653*** | | ***0.254*** | | ***0.804*** | | ***0.642*** | |
| **Place of residence** |  |  |  |  |  |  |  |  |  |
| City | 101(35.4) | 55.56[50-66.67] | 143.64 | 43.75[25-62.5] | 64.63 | 55.56[50-63.89] | 149.85 | 45.83[29.17-61.81] | 133.60 |
| Village | 169(59.3) | 55.56[50-66.67] | 140.12 | 50[31.25-68.75] | 72.02 | 55.56[44.44-66.67] | 136.66 | 54.17[39.24-69.44] | 147.05 |
| Refugee Camp | 15(5.3) | 63.89[52.78-79.17] | 171.20 | 56.25[45.31-62.5] | 85.00 | 63.89[56.95-70.83] | 168.30 | 56.25[32.99-74.31] | 160.67 |
|  | ***pValue*** | ***0.368*** | | ***0.452*** | | ***0.203*** | | ***0.298*** | |
| **Household Income** |  |  |  |  |  |  |  |  |  |
| Low: Less than 400 JD | 145(50.9) | 55.56[50-66.67] | 139.78 | 43.75[25-56.25] | 61.05 | 55.56[44.44-66.67] | 132.32 | 45.83[29.17-62.5] | 134.20 |
| Moderate: Between 400-1000 JD | 119(41.8) | 55.56[50-72.22] | 142.89 | 53.13[31.25-70.31] | 78.15 | 61.11[50-66.67] | 154.56 | 58.33[37.5-69.44] | 148.49 |
| High: More than 1000 JD | 20(7) | 61.11[48.61-81.94] | 159.93 | 56.25[37.5-93.75] | 96.00 | 50[34.72-65.28] | 144.53 | 61.81[35.42-79.86] | 167.05 |
|  | ***pValue*** | ***0.583*** | | ***0.016*** | | ***0.085*** | | ***0.141*** | |
| **BMI category** |  |  |  |  |  |  |  |  |  |
| Underweight / Normal | 52(18.2) | 61.11[44.44-70.83] | 141.00 | 50[32.81-68.75] | 76.63 | 61.11[50-66.67] | 146.73 | 45.83[36.11-69.1] | 142.81 |
| Overweight | 129(45.3) | 61.11[50-70.83] | 144.70 | 50[31.25-67.19] | 74.70 | 55.56[50-65.28] | 150.01 | 54.17[45.83-69.44] | 151.46 |
| Obese | 104(36.5) | 55.56[50-66.67] | 141.89 | 43.75[18.75-59.38] | 61.64 | 55.56[44.44-66.67] | 132.44 | 45.83[29.86-64.58] | 132.60 |
|  | ***pValue*** | ***0.949*** | | ***0.154*** | | ***0.244*** | | ***0.220*** | |
| **Disease Activity** |  |  |  |  |  |  |  |  |  |
| Inactive | 124(43.5) | 61.11[50-72.22] | 168.60 | 56.25[37.5-68.75] | 79.40 | 61.11[50-66.67] | 161.77 | 59.72[45.83-69.44] | 163.15 |
| Low to moderate | 98(34.4) | 55.56[50-66.67] | 117.75 | 50[31.25-62.5] | 71.32 | 58.34[50-66.67] | 130.96 | 52.78[36.46-68.06] | 131.83 |
| High | 61(21.4) | 55.56[34.72-66.67] | 126.88 | 21.88[12.5-59.38] | 50.09 | 50[38.89-65.28] | 119.54 | 40.97[29.17-64.58] | 115.34 |
|  | ***pValue*** | ***<0.001*** | | ***0.004*** | | ***0.001*** | | ***<0.001*** | |
| **Treatment Status** |  |  |  |  |  |  |  |  |  |
| Regular treatment | 239(83.9) | 55.56[50-66.67] | 144.82 | 50[25-62.5] | 69.65 | 55.56[50-66.67] | 147.00 | 52.78[37.5-69.44] | 147.10 |
| non formal treatment | 46(16.1) | 63.89[38.89-73.61] | 133.53 | 43.75[29.69-68.75] | 71.84 | 52.78[38.89-66.67] | 122.22 | 48.61[29.17-61.46] | 121.72 |
|  | ***pValue*** | ***0.391*** | | ***0.815*** | | ***0.058*** | | ***0.055*** | |
| **Duration of disease (years)** |  |  |  |  |  |  |  |  |  |
| <1 | 9(3.2) | 58.33[52.78-66.67] | 167.39 | 46.88[35.94-57.81] | 71.33 | 61.11[55.56-72.23] | 177.83 | 60.42[53.13-73.26] | 187.39 |
| 1-3 years | 73(25.6) | 55.56[50-66.67] | 145.89 | 53.13[31.25-62.5] | 73.89 | 61.11[50-66.67] | 149.86 | 53.47[36.46-66.67] | 145.25 |
| 4-5 years | 52(18.2) | 55.56[48.61-66.67] | 136.44 | 46.88[23.44-64.06] | 67.38 | 61.11[54.17-66.67] | 147.65 | 45.83[34.38-69.44] | 138.47 |
| >5 years | 150(52.6) | 55.56[50-72.22] | 141.46 | 43.75[25-62.5] | 69.24 | 50[44.44-66.67] | 135.01 | 52.78[37.5-68.75] | 139.86 |
|  | ***pValue*** | ***0.735*** | | ***0.928*** | | ***0.283*** | | ***0.382*** | |
| **Total number of Comorbid diseases** |  |  |  |  |  |  |  |  |  |
| zero | 91(31.9) | 61.11[55.56-72.22] | 157.85 | 56.25[37.5-75] | 82.81 | 61.11[50-66.67] | 144.04 | 61.11[37.5-69.44] | 150.54 |
| One Comorbid Disease | 69(24.2) | 61.11[50-77.78] | 146.73 | 50[25-68.75] | 72.47 | 61.11[50-66.67] | 155.22 | 54.17[37.5-69.44] | 150.50 |
| two Comorbid Disease | 53(18.6) | 55.56[44.44-61.11] | 125.56 | 37.5[18.75-56.25] | 55.24 | 55.56[44.44-66.67] | 149.92 | 45.83[29.17-54.17] | 136.21 |
| Three Comorbid Disease | 37(13) | 55.56[38.89-58.33] | 107.24 | 50[31.25-56.25] | 69.17 | 50[44.44-63.89] | 122.50 | 52.78[29.86-64.58] | 127.23 |
| ≥ 4 Comormid Disease | 35(12.3) | 55.56[50-72.22] | 161.26 | 37.5[25-56.25] | 60.54 | 50[44.44-61.11] | 127.40 | 51.39[36.11-69.44] | 135.56 |
|  | ***pValue*** | ***0.006*** | | ***0.076*** | | ***0.232*** | | ***0.501*** | |
| **Total number of medication** |  |  |  |  |  |  |  |  |  |
| 1-3 medications | 41(14.4) | 61.11[50-72.22] | 163.29 | 50[37.5-75] | 79.57 | 61.11[38.89-66.67] | 152.68 | 45.83[37.5-76.39] | 153.06 |
| 4-6 medications | 122(42.8) | 55.56[50-66.67] | 135.97 | 50[31.25-67.19] | 73.74 | 55.56[50-66.67] | 142.30 | 52.78[37.5-66.67] | 143.68 |
| ≥7 | 122(42.8) | 55.56[50-66.67] | 143.21 | 43.75[25-62.5] | 64.25 | 55.56[48.61-66.67] | 140.45 | 52.78[30.56-69.44] | 138.94 |
|  | ***pValue*** | ***0.180*** | | ***0.261*** | | ***0.701*** | | ***0.631*** | |
